# The rs10993994 in the proximal *MSMB* promoter region is a functional polymorphism in Asian Indian subjects

**DOI:** 10.1186/s40064-015-1164-7

**Published:** 2015-07-28

**Authors:** Deepa R Mhatre, Smita D Mahale, Mohammed I Khatkhatay, Swati K Achrekar, Swapna S Desai, Dhanashree D Jagtap, Jayesh V Dhabalia, Hemant B Tongaonkar, Sucheta P Dandekar, Anand M Varadkar

**Affiliations:** Department of Biochemistry and Clinical Nutrition, Seth G.S. Medical College and K.E.M Hospital, Parel, Mumbai, 400012 India; Division of Structural Biology, National Institute for Research in Reproductive Health, Jehangir Merwanji Street, Parel, Mumbai, 400012 India; Department of Molecular Immunodiagnostics, National Institute for Research in Reproductive Health, Jehangir Merwanji Street, Parel, Mumbai, India; Department of Urology, Seth G.S. Medical College and K.E.M Hospital, Parel, Mumbai, India; Department of Genitourinary Oncology, Tata Memorial Hospital, Parel, Mumbai, India

**Keywords:** PCa, BPH, RFLP, Novel variants

## Abstract

**Background:**

The microseminoprotein gene encoding prostate secretory protein of 94 amino acids (PSP94) harbours a potential risk allele (rs10993994) for prostate cancer (PCa) in its promoter region. However, studies on rs10993994 have been sparse in Asian Indians.

**Methods:**

The present study recruited a sample population of 44 benign prostatic hyperplasia patients, 33 PCa patients and 60 healthy participants, of which, participants without other confounding risk factors for PCa were retained. The serum PSP94 (sPSP94) levels were measured by a serum-based ELISA in an earlier study. A novel RFLP technique was developed to screen for rs10993994 which was validated with direct sequencing.

**Results:**

Sequencing showed additional 4 SNPs (rs41274660, rs141211965, rs12770171, rs10669586) and 2 novel variants (GenBank accession nos. KM265191 and KM265192). In silico DNA topographical studies predicted that KM265192 would have higher cleavage intensity and more accessibility for binding of transcription factors. Even though, similar frequencies were observed for all the variants in all the three study groups, the risk allele ‘T’ (rs10993994) was seen to be associated with reduced PSP94 expression both at mRNA and protein level. Further, mRNA expression as studied by real-time PCR correlated positively with sPSP94 levels. Interestingly, CC genotype of rs10993994 showed highest sPSP94 levels in all the three study groups and was associated with Gleason score ≤7 in PCa patients. In contrast, TT genotype of rs10993994 was associated with lesser sPSP94 levels and with aggressiveness of PCa.

**Conclusion:**

rs10993994 was found to be a functional SNP in the studied Asian Indian population.

## Background

Worldwide, the incidence rate of prostate cancer (PCa) differs as per ethnicity or location. Although these rates are higher in developed regions, they are lowest in Asian countries like India (GLOBOCAN 2012). However, the low incidence rates of PCa in India have been attributed to the lack of primary screening as well as awareness as also to insufficient documentation (Zeigler-Johnson et al. 2008). So far, several single nucleotide polymorphisms (SNPs) have been identified as predisposition loci for PCa (Kote-Jarai et al. 2011). Among these SNPs, genome-wide association studies (GWAS) have identified risk allele rs10993994 (g.−57C>T), in the promoter region of the microseminoprotein (*MSMB*) gene to be associated with PCa risk (Thomas et al. 2008; Eeles et al. 2008). Numerous studies have replicated the association of the rs10993994 SNP with PCa risk in varied ethnicities and established the risk allele as a causal variant for PCa (Kote-Jarai et al. 2008; Chang et al. 2009, 2011; Lou et al. 2009).

This *MSMB* gene codes for prostate secretory protein of 94 amino acids (PSP94), a predominant protein secreted by the prostate and a strong candidate biomarker for PCa (Nam et al. 2006; Waters et al. 2010). Our earlier study has also reconfirmed the differentiating ability of serum PSP94 (sPSP94) between PCa and benign prostatic hyperplasia (BPH) (Mhatre et al. 2014). Furthermore, the sPSP94 levels have been shown to be significantly associated with the rs10993994 genotype (Chang et al. 2009; Waters et al. 2010; Xu et al. 2010a, b; Haiman et al. 2013) and both hold potential as biomarkers for PCa risk. Till date, there have been 30 SNPs reported in the dbSNP (http://www.ncbi.nlm.nih.gov/projects/SNP/snp_ref) corresponding to the *MSMB* proximal promoter region of which rs10993994 is well established as a susceptibility locus for PCa. However, there are few reports which suggest that this SNP has not been found to be a predisposing factor for benign prostatic hyperplasia (BPH) development (Ho et al. 2012; Ban and Yoo 2014). Further, the *MSMB* promoter region has recently been studied extensively; using in vitro analysis and a ~370 bp region has been shown to harbour the core promoter and two negative regulatory elements. Additionally, several putative transcription factor binding sites (TFBS) have also been allocated within the 530 bp proximal promoter region (Lou et al. 2012). Hence, delineation of the *MSMB* promoter region can lead to better understanding of the genetic variations associated with the sPSP94 levels and PCa/BPH risk.

Subsequently, it would be important to understand the contribution of genetic disparity in the *MSMB* gene promoter towards development of PCa and BPH among Asian Indians. Hitherto, there has been only one report associating the rs10993994 causal variant with PCa risk in Asian Indians (Ahn et al. 2011). Notably, no data is available for the association of this SNP with PSP94 expression in Asian Indians. Thus the aim of the present study was to understand the contribution of genetic variants of the *MSMB* gene promoter region towards altered gene expression and risk of prostate disease in Asian Indian subjects.

## Methods

### Study participants

The present study had received approval from the Clinical Ethics Committees of Seth G. S. Medical College and K.E.M Hospital Mumbai, India and Tata Memorial Hospital, Mumbai, India and the studies were performed according to the Declaration of Helsinki (WMA 2013). The participants originated from Mumbai, a cosmopolitan city of India and hence represent ethnic variation. A fair representation of different Indian ethnicities like Marathi, Bengali, Oriya, Dravidian, Gurjar and Konkani was seen in the study participants (P > 0.05). These Asian Indian men presenting with lower urinary tract symptoms (LUTS) and either an abnormal digital rectal examination (DRE) finding or a sPSA level above 4 ng/ml or both, were invited to participate in this retrospective study. Histologically confirmed cases of both BPH (n = 44) and PCa (n = 33) were selected. For the control group, healthy volunteers (n = 60), asymptomatic for prostatic diseases, were recruited from Community Health Check-up Centres. Written informed consent was obtained from all these participants (n = 137). Since the study was conducted in hospital settings and involved biopsy-confirmed cases, the probability of getting false positive was lower. Hence less than 5 patients were required to have a 90% chance of detecting, as significant at 5% level, an increase in positive biopsy from 10% in the control group to 90% in the PCa group. The inclusion of more than 30 participants in all the three study groups, BPH, PCa and healthy, would therefore allot more than sufficient power to our study, for detecting statistically significant differences. (https://www.sealedenvelope.com/power/binary-superiority/). Whole blood sample (1 ml) was collected from each of the participants, and stored at −80°C till analysed. Prostate tissue (200 mg) was obtained from the study population during radical prostatectomy (for PCa patients, n = 15) or TURP procedure (for BPH patients, n = 20) during necessary surgical intervention. Representative samples from the tissue samples collected were evaluated histologically to ascertain the status of the tissues used in this study. Out of these, five histologically tested healthy tissue samples were identified, to be used for tissue normalisation. The rest of the prostate tissue was snap-frozen in liquid nitrogen and stored at −80°C until further use. All participants for this present study were also part of our earlier study (Mhatre et al. 2014). To understand the effect of genetic variations alone on the prostate pathophysiology, we retained participants (n = 112) who were without confounding risk factors for PCa, like age, hypertension, diabetes, Body Mass Index (BMI) or smoking/tobacco chewing/drinking habits (Ganesh et al. 2011). Similarly, participants with acute prostatitis or previous history of PCa or a family history of PCa had been excluded from the present study.

### Levels of sPSA and sPSP94

The total sPSA and sPSP94 levels were noted from our earlier study (Mhatre et al. 2014), for all the samples. The sPSA levels had been measured using chemiluminiscentmicroparticle immunoassay (CMIA), Abbott Architect. The sPSP94 levels were measured using an in-house sandwich ELISA as described earlier (Mhatre et al. 2014).

### Amplification of the *MSMB* proximal promoter region

Genomic DNA extracted from 250 μl whole blood (n = 112) using a commercial kit (Axygen Biosciences, CA, USA) was used to amplify a region of the *MSMB* gene from nucleotides at positions −565 to +25 (590 bp) by polymerase chain reaction (PCR) using published primers with minor modifications (Chang et al. 2009). Amplification reactions contained 50 ng of template gDNA, 10 μl of 5× PCR buffer (Promega, Madison, USA), 3 μl of MgCl_2_, 10 mM dNTP mix, 20 μM primers and 2.5 U GoTaq Polymerase (Promega, Madison, USA) in a total volume of 50 μl. The PCR products were subjected to clean-up (AxyPrep PCR cleanup kit, GenAxy, HP, India).

### Restriction fragment length polymorphism (RFLP) for detecting SNP rs10993994 of *MSMB* gene

A RFLP analysis was developed to screen the rs10993994 polymorphism in the *MSMB* gene. The presence of C allele at rs10993994 position provides a recognition site (GA**C**GTC) for the restriction enzyme *Aat*II whereas presence of T allele results in loss of this restriction site. Restriction digestion was carried out at 37°C for 2 h using 5 μl PCR clean-up product and 10 IU of the *Aat*II enzyme (New England BioLabs, Ipswich, MA, USA) in a total volume of 1× reaction buffer (New England BioLabs, Ipswich, MA, USA). The products were subjected to (1.5%) agarose gel electrophoresis and visualised under UV light.

### Direct sequencing

Since the RFLP technique for rs10993994 was novel, hence sequence confirmation was carried out for all the samples. Genotyping of the proximal *MSMB* promoter region was performed by direct sequencing of the PCR amplicon (590 bp). The eluant containing 120 ng DNA was subjected to direct DNA sequencing (SAF labs, Mumbai, India). Sequences were compared to a reference sequence (NC_000010.11). Variants were reconfirmed after repeat sequencing.

### DNA topography

Nucleotide changes can affect the three-dimensional (3D) molecular structure of DNA. This can in turn bring about differences in transcription factor binding. DNA topographical changes occurring due to the *MSMB* promoter variants found in the sample population were assessed by ranking the structure-change value. These were revealed by the predicted hydroxyl radical cleavage pattern for the DNA sequence of the promoter region using the OH Radical Cleavage Intensity Database (Greenbaum et al. 2007). The effect of the nucleotide change on the structural profile was quantified in terms of Euclidean distance, which is a measure to compute the average structure change (Parker et al. 2009).

### Quantitative real time PCR

RNA was extracted using the Trizol reagent (Invitrogen, Carlsbad, CA) as per manufacturer’s instructions from 50 to 100 mg of prostate tissue. All the experimental work was carried out under RNase-free environment using RNA-grade labware and reagents. The first-strand cDNA was synthesized from 2 μg of total RNA using the ThermoScript™ RT-PCR System for First-Strand cDNA Synthesis Kit (Invitrogen, Carlsbad, CA, USA) as per manufacturer’s instructions. The primers for the PSP94 transcript, *MSMB1*, were as mentioned by Pathak et al. (2010) and primers for the gene of the house-keeping protein β-actin, (*ACTB*) were as mentioned by Cai et al. (2007). These primers were used for amplification of the cDNA by real-time PCR in duplicate reactions using SYBR green chemistry in a CFX96 qPCR system (BioRad, Hercules, CA, USA). The difference in the C_t_ values of *MSMB1* in the test (BPH or PCa) and the calibrator sample (healthy) was noted as ΔC_t_ (*MSMB1*, Healthy—BPH or PCa). Similarly, the ΔC_t_ (*ACTB*, Healthy—BPH or PCa) was also noted for all samples. E_*MSMB1*_ and E_*ACTB*_, the amplification efficiencies of the qPCR reaction were calculated and the relative gene expression was determined as a ratio, by the Pfaffl method.

### Statistical analysis

The baseline characteristics of the study participants were compared by ANOVA or unpaired *t* test, as applicable. Genotype frequencies were calculated for all the three study groups—PCa, BPH and healthy. Frequency distribution of the genotypes was analyzed by Chi square (χ^2^) test. Concordance with Hardy–Weinberg equilibrium was calculated with a χ^2^ analysis (Rodriguez et al. 2009). Relation between sPSP94 expression and genotypes was compared by one-way ANOVA and Dunn’s post hoc multiple comparisons test. Statistical analysis was performed with Statistics Package for Social Sciences (SPSS) for Windows, version 16 (SPSS Inc., Chicago, IL, USA). Graphical representations and Pearson’s correlation studies were carried out using GraphPad Prism Version 5.0 for Windows. *P* ≤ 0.05 was considered statistically significant for all studies.

## Results

### Clinical parameters of study subjects

Various parameters such as age, Gleason score (GS), sPSA and sPSP94 levels were assessed to study their association with prostate pathophysiology. All participants were age-matched. In order to focus on the level of genetic disparity in different prostate conditions, we selected biopsy-confirmed PCa (n = 32)/BPH (n = 30) men and healthy controls (n = 50) without other confounding risk factors (Table 1). The sPSA levels in the 0–4 ng/ml range of BPH patients were statistically different from those of the healthy participants. Interestingly, the sPSP94 levels of BPH patients were found to be statistically different from those of PCa patients as well as healthy participants (Table 1).

**Table 1:** Baseline characteristics of the study participants

Factor	Characteristic	Healthy		BPH		PCa		*P* value
		n	Mean (SE)	n	Mean (SE)	n	Mean (SE)	
Age, years	–	50	63.22 (1.01)	30	65.57 (1.66)	32	66.06 (1.61)	ns
DRE findings	Normal	–	–	11	–	04	–	–
	Non-palpable	–	–	19	–	09	–	–
	Nodule	–	–	–	–	19	–	–
Biopsy findings	PCa	–	–	00	–	32	–	–
	BPH	–	–	30	–	00	–	–
Gleason score	GS ≤7	–	–	–	–	16	–	ns
	GS >7	–	–	–	–	16	–	ns
History of hypertension	Yes	21	–	14	–	15	–	ns
	No	29	–	16	–	17	–	ns
History of diabetes	Yes	22	–	15	–	14	–	ns
	No	28	–	15	–	18	–	ns
Body mass index	≥25	27	–	16	–	16	–	ns
	<25	23	–	14	–	16	–	ns
Smokers^c^	Yes	18	–	10	–	13	–	ns
	No	32	–	20	–	19	–	ns
Chewers^d^	Yes	25	–	19	–	19	–	ns
	No	25	–	11	–	13	–	ns
Alcohol drinkers	Yes	28	–	16	–	14	–	ns
	No	22	–	14	–	18	–	ns
sPSA ng/ml	0–4	50	0.98 (0.12)^a^	17	2.05 (0.27)^a^	03	1.86 (0.84)	<0.05
	>4–10	–	–	06	7.45 (0.67)	04	7.33 (1.17)	ns
	>10	–	–	07	49.78 (30.42)	25	87.99 (21.92)	ns
sPSP94 ng/ml		50	18.07 (1.06)^b^	30	28.17 (2.44 )^a,b^	32	19.68 (1.98)^a^	<0.05

^a,b^Denote significantly different groups (*P* < 0.05).

^c^Implies cigarette and/or bidi smoking.

^d^Implies chewing betel leaf, tobacco, and tobacco products.

### RFLP analysis of the rs10993994 genotype

**Fig. 1:**
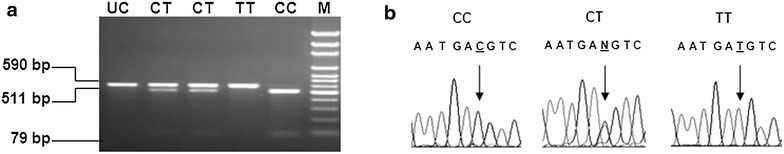
Genotyping for rs10993994. **a** agarose gel picture showing the different rs10993994 *MSMB* promoter polymorphism genotypes. *Lane 1: UC* Undigested PCR clean-up product; *Lanes 2–5:* digested PCR clean-up products; *Lanes 2 and 3: CT* three fragments of 590, 511 and 79 bp indicating heterozygous CT genotype; *Lanes 4: TT* uncut fragment of 590 bp indicating homozygous TT genotype; *Lane 5: CC* two fragments of 511 and 79 bp indicating homozygous CC genotype; *Lane 6: M* 100 bp DNA ladder. **b** electropherogram of 5 → 3′ strand showing corresponding sequence. *C*, *N*, *T* indicated by an *arrow*, correspond to CC, CT and TT genotypes at position −57 respectively.

RFLP analysis, for rs10993994 SNP, revealed three different patterns for the three genotypes (Fig. 1a). Homozygous CC genotype retained the restriction site (GA**C**GTC) for enzyme *Aat*II, whereas homozygous TT genotype could not be restriction-digested. Thus, heterozygous CT genotype showed presence of all three fragments sizes 590, 511 and 79 bp.

### Frequency distribution

Results of RFLP were validated through sequencing of all samples (n = 112). Individual genotype was identified through colour code as seen in the representative electropherogram (Fig. 1b). It was observed that the results of RFLP for the rs10993994 genotype positively correlated with the genotype as obtained by direct sequencing (r = 0.9737, P < 0.0001). Frequency distribution of rs10993994 SNP in healthy subjects (n = 50) was 18% for CC genotype, 48% for CT genotype and 34% for TT genotype. For BPH patients (n = 30), prevalence of CC genotype was 16.7%, of CT genotype was 33.3 and 50% for TT genotype. In case of PCa patients (n = 32), the CC genotype was found to be 9.4%, the CT genotype was 56.3% whereas TT genotype was 34.4%. No statistically significant differences were observed in the frequency distribution of all the three genotypes among the three study groups (Table 2).

**Table 2:** List of known and novel variants identified by direct sequencing in the *MSMB* proximal promoter region

Variant^a^	Promoter position^b^	Healthy group genotype counts (n = 50)			BPH genotype counts (n = 30)			PCa genotype counts (n = 32)			*P* value
		HomWT^c^	Het^d^	HomVar^e^	HomWT^c^	Het^d^	HomVar^e^	HomWT^c^	Het^d^	HomVar^e^	
rs41274660:T>G	−20	50	00	00	30	00	00	31	01	00	ns
rs10993994:C>T	−57	09	24	17	05	10	15	03	18	11	ns
rs141211965:C>T	−80	49	01	00	30	00	00	32	00	00	ns
rs12770171:C>T	−239	28	19	03	15	13	02	24	06	02	ns
rs10669586:indelCT	−331	49	01	–	24	06	–	28	04	–	ns
KM265191:C>T^f^	−176	50	00	00	30	00	00	31	01	00	ns
KM265192:A>G^f^	−343	49	01	00	29	01	00	32	00	00	ns

^a^Major/Minor allele.

^b^Position from start of exon1.

^c^Homozygous wild type.

^d^Heterozygous.

^e^Homozygous variant.

^f^Novel finding—rare variant.

Additionally, the proximal promoter region showed presence of four other known SNPs. None of the five SNPs had significant differences in their frequency distributions. Interestingly, two novel rare variants were also observed. The first variant, was located at position −176 on the *MSMB* promoter and was seen in a PCa patient. As well, the second variant, was located at position −343 on the *MSMB* promoter (Table 2).

### DNA topography of the *MSMB* promoter region

**Fig. 2:**
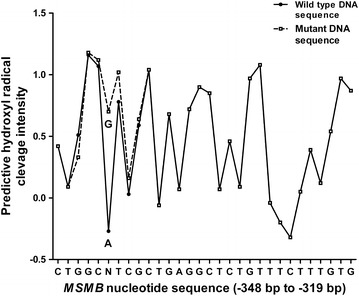
DNA topographical changes at position −343. Comparison of the predicted hydroxyl radical cleavage intensity corresponding to the nucleotide sequence of the wild-type and novel variant at position −343 in the *MSMB* gene promoter region.

All the *MSMB* promoter DNA sequences harboring the five SNPs as well as the two novel rare variants were compared with the wild type sequence (NC_0.000010.11) to investigate whether these single nucleotide changes could modify the 3D molecular structure of the DNA and thereby alter the transcription process. Of these seven variants, we observed that the novel rare variant (G allele), located at position −343 resulted in a structural change value greater than 0.8 (Table 3). The DNA with the G allele (0.70) at this position is more accessible for binding of transcription factors compared with the wild type A allele (−0.27) (Fig. 2).

**Table 3:** Predicted increase in cleavage intensities due to presence of the variant nucleotide

*MSMB* variant^a^	Promoter position^b^	DNA structural change value
rs41274660:T>G	−20	0.66
rs10993994:C>T	−57	0.07
rs141211965:C>T	−80	0.02
rs12770171:C>T	−239	0.39
rs10669586:indelCT	−331	0.51
KM265191:C>T^c^	−176	0.02
KM265192:A>G^c^	−343	*0.97*

^a^Major/Minor allele.

^b^Position from start of exon1.

^c^Novel finding—rare variant.

### Association of rs10993994 genotype of *MSMB* gene with sPSP94 mRNA (*MSMB1*) expression

**Fig. 3:**
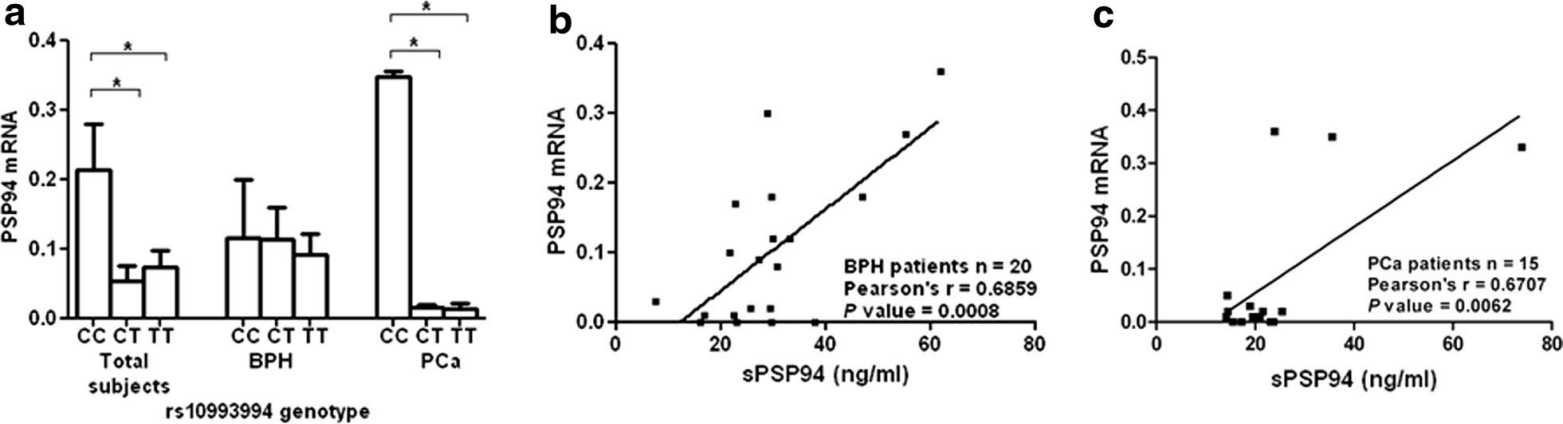
Association of rs10993994 with PSP94 RNA expression. **a** the *bar diagram* showing the comparison of PSP94 mRNA expression levels in prostate (mean ± SEM) among the rs10993994 genotypes, in the study population. The statistical analysis was carried out by one-way ANOVA and Dunn’s post hoc multiple comparisons test, and the *brackets* accompanying *asterisks* represent significantly different groups. **b** correlation of sPSP94 levels with PSP94 prostatic mRNA expression in BPH patients. **c** correlation of sPSP94 levels with PSP94 prostatic mRNA expression in PCa patients. Statistical significance is at *P* < 0.05.

We observed that the *MSMB1* expression was lower in TT genotype as compared to CC genotype. Further, the *MSMB1* in PCa patients having CC genotype showed statistically higher expression than in PCa patients with CT or TT genotype (Fig. 3a). Also it was interesting to note that the *MSMB1* mRNA expression showed moderate positive correlation with the sPSP94 protein levels in both BPH (Fig. 3b) as well as PCa patients (Fig. 3c).

### Association of rs10993994 genotype of *MSMB* gene with sPSP94 levels

**Fig. 4:**
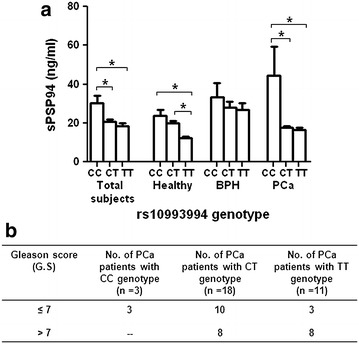
Association of rs10993994 with sPSP94 levels and Gleason score. **a** the *bar diagram* showing the comparison of sPSP94 levels (mean ± SEM) among the rs10993994 genotypes, in the study population. The statistical analysis was carried out by one-way ANOVA and Dunn’s post hoc multiple comparisons test, and the *brackets* accompanying *asterisks* represent significantly different groups. Statistical significance is at *P* < *0.05.* **b** sub classification of rs10993994 genotypes in PCa patients on the basis of Gleason score.

To analyze effect of the rs10993994 genotype of the *MSMB* gene on secreted levels of the PSP94 protein, in our sample cohort, we segregated sPSP94 values on the basis of genotype in all study participants. The TT genotype showed the minimum sPSP94 levels among healthy, BPH and PCa groups and the sPSP94 levels of TT genotype in the healthy group were significantly different from CC and CT genotypes (*P* < 0.05). Also, we observed that CC genotype showed maximum levels of sPSP94 among all three groups. Further, only sPSP94 levels of CC genotype of the PCa group were significantly different from the CT and TT genotypes (*P* < 0.05) (Fig. 4a). It was interesting to note that, all PCa patients with CC genotype had a GS ≤7. On the other hand, 7 out of the 10 PCa patients with TT genotype had a GS >7 (Fig. 4b).

## Discussion

Human prostate cancer is a multifactorial disease that involves interaction between genetic susceptibility and other factors like age, family history and lifestyle (Crawford 2003). In a recent study of Asian Indian men, age, BMI and hypertension were seen to be the predominant determinants for PCa (Ganesh et al. 2011). Further, worldwide, among all the genetic susceptibility loci for PCa, *MSMB* promoter variant, rs10993994, has emerged as a predisposition locus for risk of the disease (Thomas et al. 2008; Eeles et al. 2008; Chang et al. 2009; Lou et al. 2009; Pomerantz et al. 2010). However, another study suggests that rs10993994 may not be a PCa risk SNP in men of Chinese Han origin (Huang et al. 2009). In the current study, the sample population was selected to have no other confounding risk factors for PCa. Interestingly we observed that the frequency of this SNP was similar in healthy participants as well as BPH and PCa patients in this population. This could be attributed to lesser genetic contribution towards PCa or to the different geographical and racial representation in the studied population. Other than the current study, a report on the significance of rs10993994, which included 122 PCa patients from North India, suggested that this SNP was associated with risk for metastatic PCa (Ahn et al. 2011). Hence, data generation through more replicative studies of larger sample size in Asian Indians are therefore required for asserting rs10993994 as a predisposition locus in Asian Indians. This is necessary since no consensus has been met regarding association of rs10993994 with clinicopathologic features of PCa. Whereas a higher frequency of the T risk allele has been found in patients with more aggressive PCa (Thomas et al. 2008; Kader et al. 2009), this has not been observed in other studies (Xu et al. 2008; Waters et al. 2009).

The *MSMB* gene encodes, PSP94, whose decreased serum levels have been established as a detection and prognostic marker for PCa (Nam et al. 2006; Reeves et al. 2006). Further, all the functional studies consistently showed that the T allele at rs10993994 was associated with lower sPSP94 levels as compared to the C allele (Waters et al. 2010; Xu et al. 2010b; Haiman et al. 2013). Interestingly, this is attributed to the disruption of a cAMP response element-binding protein (CREB) binding site, in the risk allele (Lou et al. 2009). Similarly, other TFBS and regulatory elements on the proximal promoter region of *MSMB* have been shown to alter the gene expression in vitro (Lou et al. 2012). This suggests that the proximal promoter region of the *MSMB* gene could hold the potential in altering the PSP94 gene expression. Hence in the present study, we screened the entire proximal *MSMB* promoter in our study population. We observed presence of five known variants of which, SNP rs10669586 shows deletion at position −331. The rs12770171 variant at position −239, which is in linkage disequilibrium with rs10993994 (Kote-Jarai et al. 2010) was also observed to have similar frequency distribution among the three study groups. The variants at position −20 and position −80, interestingly showed presence of a heterozygous condition, each in a single study participant. Since the data regarding occurrence of these SNPs in Asian Indians is not known, therefore, there is a need to further screen the *MSMB* promoter region to understand the significance of these variants on PSP94 expression.

Concurrently, we also report for the first time, evidence of two novel rare genetic variants in this region. In case of the novel variant at position −176, a heterozygous genotype was observed in a single PCa patient. Whereas the heterozygous novel variant at position −343, was observed in a BPH patient and a healthy participant. It should be interesting to study the rare novel variant at both these positions by in vitro analyses to access the functional significance of these variants in the *MSMB* core promoter region. Nonetheless, our in silico analysis suggests that, the DNA sequence harbouring the G-allele, located at position −343, could result in stearic changes in structure, leading to enhanced accessibility of the TFBS at this position (Lou et al. 2012), compared to the A-allele. This variant is located in one of the negative regulatory elements of the *MSMB* promoter harbouring the EBOX-CREB two TFBS module. Hence it would be fascinating to study the altered binding of the transcription factors at position −343 in the presence of G-allele. So far, the functional studies of the *MSMB* proximal promoter has only been restricted to rs10993994. In our in silico analysis, the cleavage intensity predicted for rs10993994 does not suggest significant difference in the DNA–protein binding affinity for the C- and T-alleles. However, it has been shown that the presence of T- allele at rs10993994, results in upto 13% decrease in MSMB promoter activity as compared to the C-allele (Chang et al. 2009). Since this SNP is in a CREB binding site, this decrease in activity could be due to the effect of the marginal decrease in DNA affinity on the CREB activation cascade like protein kinase A (PKA) phosphorylation (Richards et al. 1996).

In our current study, we observed that the rs10993994 genotype is associated with altered levels of both the *MSMB1* mRNA as well as the sPSP94 levels. Firstly, it was seen that the TT genotype leads to lesser mRNA expression than CC genotype, in both BPH as well as PCa patients. This has also been reported earlier in histologically normal prostate tissue as well as in PCa tissue (Pomerantz et al. 2010). Additionally, the mRNA expression positively correlated with the sPSP94 levels in both BPH as well as PCa patients of our study population, suggesting that, the sPSP94 levels are indicator of the prostatic expression of the protein. Subsequently, the sPSP94 levels of BPH as well as PCa patients with TT genotype were significantly lower as compared to CC genotype. However, a similar trend was also observed in healthy controls. Consequently, irrespective of the risk for PCa/BPH, the T allele of rs10993994 was consistently seen to be associated with lower sPSP94 levels. These findings are in agreement with three individual large studies which also show that the T allele is associated with lesser sPSP94 levels, in PCa cases as well as controls (Waters et al. 2010; Xu et al. 2010b; Haiman et al. 2013). Nevertheless, the association of rs10993994 with both the altered sPSP94 expression and PCa risk has been established earlier (Xu et al. 2010a; Haiman et al. 2013). Thus these contradictory observations open forum on the question of the biological significance of influence of rs10993994 on sPSP94 levels and PCa risk, since this SNP also alters the PSP94 expression in healthy men.

Concomitantly, on sub-classification of the PCa subjects based on Gleason score (GS), it was interesting to note that our study population has eleven PCa patients carrying the TT genotype and eight of these patients had a GS >7, suggesting the probable role of this genotype towards aggressiveness of the cancer. This is similar to an earlier study in Chinese Han population, where the T allele was found to be associated significantly with increased PCa risk in subjects with GS >7 (Xu et al. 2010a). Moreover, the CC genotype was the least common in PCa study population and the subjects (n = 3) with CC genotype had a GS of ≤7 which indicates its probable protective ability against PCa. Thus the rs10993994 SNP may not have a role in occurrence of, but in aggressiveness of PCa.

## Conclusions

Through our study we conclude that rs10993994 affects PSP94 expression at mRNA and protein level irrespective of the physiological state. Moreover since our sample population was without other confounding risk factors for PCa, we could concentrate our study exclusively on the effect of the rs10993994 genotype on PCa/BPH risk. Results suggest that rs10993994 may not be solely contributing towards development of PCa/BPH but could be a marker of PCa aggressiveness. These preliminary findings need to be validated in larger sample size as well as in different Indian ethnicities.

## Abbreviations

*MSMB*: microseminoprotein
PSP94: prostate secretory protein of 94 amino acids
PCa: prostate cancer
BPH: benign prostatic hyperplasia
SNP: single nucleotide polymorphism
sPSP94: serum PSP94
TFBS: transcription factor binding site
sPSA: serum prostate specific antigen
RFLP: restriction fragment length polymorphism
GS: Gleason score
CREB: cAMP response element-binding protein
